# Arabidopsis Mutant *bik1* Exhibits Strong Resistance to *Plasmodiophora brassicae*

**DOI:** 10.3389/fphys.2016.00402

**Published:** 2016-09-13

**Authors:** Tao Chen, Kai Bi, Zhangchao He, Zhixiao Gao, Ying Zhao, Yanping Fu, Jiasen Cheng, Jiatao Xie, Daohong Jiang

**Affiliations:** ^1^State Key Laboratory of Agricultural Microbiology, Huazhong Agricultural UniversityWuhan, China; ^2^The Provincial Key Lab of Plant Pathology of Hubei Province, College of Plant Science and Technology, Huazhong Agricultural UniversityWuhan, China

**Keywords:** *Arabidopsis thaliana*, *Plasmodiophora brassicae*, BIK1, SA, ROS, SAR, Clubroot

## Abstract

Botrytis-induced kinase1 (BIK1), a receptor-like cytoplasmic kinase, plays an important role in resistance against pathogens and insects in *Arabidopsis thaliana*. However, it remains unknown whether BIK1 functions against *Plasmodiophora brassicae*, an obligate biotrophic protist that attacks cruciferous plants and induces gall formation on roots. Here, we investigated the potential roles of receptors FLS2, BAK1, and BIK1 in the infection of *P. brassicae* cruciferous plants. Wild-type plants, *fls2*, and *bak1* mutants showed typical symptom on roots, and the galls were filled with large quantities of resting spores, while *bik1* mutant plants exhibited strong resistance to *P. brassicae*. Compared with that of the wild-type plants, the root hair and cortical infection rate of *bik1* mutant were significantly reduced by about 40–50%. A considerable portion of *bik1* roots failed to form typical galls. Even if some small galls were formed, they were filled with multinucleate secondary plasmodia. The *bik1* plants accumulated less reactive oxygen species (ROS) at infected roots than other mutants and wild-type plants. Exogenous salicylic acid (SA) treatment alleviated the clubroot symptoms in wild-type plants, and the expression of the *S*A signaling marker gene *PR1* was significantly increased in *bik1*. Both *sid2* (salicylic acid induction-deficient 2) and *npr1-1* [non-expresser of PR genes that regulate systemic acquired resistance (SAR)] mutants showed increased susceptibility to *P. brassicae* compared with wild-type plants. These results suggest that the resistance of *bik1* to *P. brassicae* is possibly mediated by SA inducible mechanisms.

## Introduction

The soil-borne obligate pathogen *Plasmodiophora brassicae* causes clubroot disease in species of *Brassicaceae*, including Arabidopsis. *P. brassicae* plunders nutrients from host roots to complete their life cycle, and causes the formation of root galls. Clubroot occurs in more than 60 countries and results in a 10–15% reduction in the yields of Cruciferae crops on a global scale (Dixon, 2009). *P. brassicae* has a complex but not completely understood infection process. A primary zoospore is released from each resting spore, which reaches the surface of a root hair and penetrates the cell wall, forming primary plasmodia in the root hair. After a number of nuclear divisions, the plasmodia cleave into zoosporangia, and the zoosporangia form clusters in the root hair or penetrate root cortex cells, in which the pathogen develops into secondary plasmodia (Kageyama and Asano, 2009). Plasmodia provoke abnormal cell enlargement and uncontrolled cell division, leading to the development of club-shaped galls on the roots and the above-ground symptoms, such as wilting, stunting, yellowing, and premature senescence compared with healthy plants (Hwang et al., 2012). Each gall contains millions of resting spores that persist in the soil for up to 15 years even in the absence of a suitable host, making it difficult to manage clubroot diseases (Donald and Porter, 2009). The resting spores of *P. brassicae* are easily transmitted to elsewhere with contaminated soil, including farm machinery, boots, grazing animal hooves, infected transplants, and surface floodwater (Donald and Porter, 2009). Crop rotation, increased soil pH, improved drainage conditions, and fungicide application provide certain protection against the disease; however, under high clubroot pressure, these measures are generally not effective (Abbasi and Lazarovits, 2006). Besides, government policies concerning human health and environmental safety have led to the restriction or deregistration of a large number of previously useful active ingredients. Genetic resistance is the most effective and economical approach to clubroot management, and several resistant cultivars against clubroot have been previously reported (Hirai et al., 2004; Rocherieux et al., 2004; Chu et al., 2014). However, new races of the pathogen can rapidly appear (Fähling et al., 2003). Using *Arabidopsis thaliana* as a model system has facilitated the application of available genetic and molecular tools and has thus advanced our understanding of this economically important plant disease (Siemens et al., 2002). In clubroot-infected Arabidopsis, it is thought that perturbation in phytohormone content plays important roles in disease development (Malinowski et al., 2012), but little has been known about which genes are involved in the plant defense response.

Salicylic acid (SA) is an important secondary phenolic metabolite in a wide range of prokaryotic and eukaryotic organisms, including plants. SA regulates a multitude of developmental processes, such as plant cell growth, seed germination and development, thermo-tolerance, respiration, stomatal aperture, fruit yield, nodulation in legumes, and leaf senescence. More importantly, SA serves as a key signaling and regulatory molecule in plant defense responses, and it is regarded as the key plant immune hormone (Spoel and Dong, 2012; Liu et al., 2015). The biosynthesis of SA on pathogen detection is essential for local and systemic acquired resistance (SAR) and the accumulation of pathogenesis-related (PR) proteins (Boatwright and Pajerowska-Mukhtar, 2013). SA has long been recognized as a central component of defense in plants against a number of biotrophic pathogens and viruses (Vlot et al., 2009). The development of disease in a susceptible host and its molecular basis has been recently examined using an Arabidopsis model (Agarwal et al., 2011). In two Arabidopsis genotypes Col-0 (susceptible) and Bur-0 (partially resistant) which were infected with the virulent *P. brassicae*, clubroot development was partially inhibited by camalexin, SA and jasmonic acid (JA) signaling pathway (Lemarié et al., 2015a,b). Exogenous SA in *B. oleracea* enhances resistance to clubroot (Lovelock et al., 2013, 2016). SA methyltransferase gene *PbBSMT* was identified from *P. brassicae*, which can methylate SA in host cells (Ludwig-Muller et al., 2015).

Basal resistance, the first step in plant defense response, involves perception through surface-localized pattern recognition receptors (PRRs) of conserved molecules characterized by pathogen-associated molecular patterns (PAMPs) or microbe-associated molecular patterns (MAMPs) (Monaghan and Zipfel, 2012). A well-known PRR is Arabidopsis receptor kinase FLS2, which recognizes a conserved 22 amino acid N-terminal sequence of the bacterial flagellin protein (flg22) (Gomez-Gomez and Boller, 2000). FLS2 serves as an excellent model to understand plant innate immune signaling, and heterotrimeric G proteins are directly coupled to the FLS2 receptor complex to regulate immunity (Macho and Zipfel, 2014; Liang et al., 2016). The extracellular leucine-rich repeat domain of FLS2 perceives flg22 and rapidly recruits another LRR receptor-like kinase called BAK1, which plays a role in brassinolide signaling (Chinchilla et al., 2007; Schulze et al., 2010). BIK1, a receptor-like cytoplasmic kinase, is directly phosphorylated through BAK1 and is associated with the FLS2/BAK1 complex in modulating PAMP-mediated signaling (Lu et al., 2010; Zhang et al., 2010). The inactivation of BIK1 causes severe susceptibility to necrotrophic fungal pathogens but enhances the resistance against a virulent strain of the bacterial pathogen *Pseudomonas syringae* pv *tomato* (Veronese et al., 2006), and *bik1* plants displayed enhanced antibiosis and antixenosis toward aphids through inducing the up-regulation of *PAD4* expression (Lei et al., 2014).

In the present study, we investigated the potential roles of the receptors FLS2, BAK1 and BIK1, which have different levels of basal resistance to the compatible strain ZJ-1 of the clubroot pathogen *P. brassicae*. We challenged these loss-of-function mutants with *P. brassicae. bik1* exhibited strong resistance to *P. brassicae*, and the root hair and cortical infections were significantly decreased. Besides, the development of *P. brassicae* was inhibited in the infected *bik1* plants. We attempted to explain the mechanism underlying the resistance of *bik1* mutants against *P. brassicae. bik1* plants were reported to have a higher basal SA level than wild type plants (Veronese et al., 2006; Lei et al., 2014), and SA suppresses the formation of clubroots in broccoli (Lovelock et al., 2013, 2016). The obtained results showed that exogenous SA treatment could alleviate the symptoms of clubroot. In the mutant line *sid2* and *npr1-1* mutants, which had blocked SA biosynthesis and were SAR-deficient respectively, clubroot symptoms were found to be clearly more severe compared with in Col-0. These findings indicate that the Arabidopsis mutant *bik1* exhibits strong resistance to *P. brassicae* possibly because SA inducible mechanisms enhance the resistance to clubroot disease. However, the *bik1 sid2* double mutant showed strong resistance to *P. brassicae*, suggesting that this resistance is possibly attributable to SA pathway and other unknown pathways.

## Materials and methods

### Plant materials, *P. brassicae* inoculation and growth conditions

*Arabidopsis thaliana* ecotype Columbia (Col-0), which was used as the wild-type control in the present study, was kindly provided by Dr. Yangdou Wei at the University of Saskatchewan, and the mutants *fls2, bik1, bak1-4, bik1 sid2* were kindly donated by Dr. Libo Shan (Texas A & M university). *sid2* was bought from the Arabidopsis Biological Resource Center. The *bik1* mutant line was further identified as a homozygous mutant. The RT-PCR results showed that the *bik1* mRNA could not be detected in homozygous mutant plants (Figure S1), suggesting that these plants represent knockout mutants at the BIK1 locus. The *bik1 sid2* double mutant line was identified as a homozygous mutant. The RT-PCR results showed that the *bik1* and *sid2* mRNA could not be detected in homozygous mutant plants (Figure S2).

*P. brassicae* strain ZJ-1 was originally isolated from a diseased plant in a rapeseed field in Zhijiang County, Hubei Province, P R China. The virulence of single-spore isolated from *P. brassicae* strain ZJ-1 was tested on the differential hosts of Williams, showing the single-spore isolate derived from race 1 (Williams, 1966; Fähling et al., 2004). Resting spores of *P. brassicae* were extracted from clubroot galls (Asano et al., 1999), surface disinfested by freshly prepared 2% chloramine-T solution at room temperature for 20 min, washed twice with sterile water, adjusted to a concentration of 1.0 × 10^7^ spores per mL, and were then stored at 4°C. *P. brassicae* was proliferated using the stored resting spores in greenhouse with rapeseed. Arabidopsis Col-0 and mutant seeds were germinated on the surface of vermiculite in small pots. The seedlings were transplanted to the soil at 14 days post-germination. After growing of 7 days in a growth chamber, plants were inoculated with 1 mL of the resting spore suspension (1 × 10^7^ spores per mL) by injecting the soil around each plant. Arabidopsis and mutant plants grown in the 50 holes plate (54 cm × 28 cm × 5 cm), each seeding planted in one hole, the wild type and mutants Arabidopsis were planted in the same holes plate to reduce errors. The plants were grown in a plant growth chamber maintained at 70% humidity and 23°C with a 16/8-h day/night cycle. For all the mutants analyzed, gene expression was verified at 21 or 28 days post inoculation. Disease severity was assessed using a scoring system of 0–4 modified from Siemens reported (Siemens et al., 2002). A score of 0 indicated no disease; 1, very small galls mainly on lateral roots that did not impair the main root; 2, small galls covering the main root and few lateral roots; 3, medium to large galls, also on the main root; and 4, severe galls on lateral root, main root or rosette, with fine roots completely destroyed. Disease index (DI) was calculated using the five-grade scale according to the formula: DI = (1n_1_ + 2n_2_ + 3n_3_ + 4n_4_) × 100/4N_t_, where n_1_–n_4_ is the number of plants in the indicated class and N_t_ is the total number of plants tested.

### ROS determination

Reactive oxygen species (ROS) production was detected using the nitroblue tetrazolium (NBT) staining method (Montiel et al., 2012; Arthikala et al., 2014). The plants grown in the soil pot for 21 days after infection were used to determine ${\text{O}}_{2}^{-}$ concentrations. The healthy roots and the galled roots were incubated for 5 h in dark at room temperature, and the roots were cleared in 90% ethanol.

### DAB staining

To estimate the produced H_2_O_2_ *in situ*, the roots were hand-sectioned using a double-edged razorblade. The sections were subsequently rapidly immersed in 1 mg/ml of 3′, 3′-diaminobenzidine (DAB) solution, vacuum infiltrated for 2 min, and incubated for 2–3 h at 25°C in darkness (Gorska-Czekaj and Borucki, 2013).

### SA and MeSA exogenous treatment

A stock solution of SA (99.5%, Sigma Aldrich, dried substance) was prepared in ethanol/ddH_2_O (v/v1:1), and diluted with sterile water at 2.5 × 10^−5^mol/L concentration for spraying (Lovelock et al., 2013). MeSA (99%, VETEC) was diluted in 10% ethanol and was sprayed at the 2.5 × 10^−5^mol/L concentration. SA and MeSA were used for three times of exogenous treatments. The first treatment was conducted 2 days prior to the inoculation, while the second treatment was carried out 2 days after inoculation, and the last was performed 10 days after inoculation. The total volume of each spraying was 2 L, and the remaining water was gently poured into the holding tray for slow absorption through the roots. Approximately 30 plants were treated for each sample (Lovelock et al., 2013).

### Quantification of *P. brassicae* DNA content in infected roots

DNA was extracted from root samples using the cetyl trimethyl ammonium bromide (CTAB) method (Allen et al., 2006). Quantitative PCR was performed on a CFX96 real-time PCR system (BioRad) using iTaq Universal SYBR Green supermix (BioRad) to quantify the *P. brassicae* target actin gene AY452179.1. Each reaction was performed with 2.5 ng of total DNA as template, and Arabidopsis actin gene AT3G18780 was used as an internal control for data normalization. Standard curves were constructed using serial dilutions of DNA extracted from the roots of Col-0 at 21 days after inoculation with *P. brassicae*, which was defined as a reference condition. Quantitative results were then expressed as the % of the *P. brassicae* mean DNA content in this reference condition (Lemarié et al., 2015a,b).

### RNA isolation and quantitative real-time PCR

Total RNA was isolated from control roots, and the roots were inoculated with *P. brassicae* using TRIZOL reagent (Invitrogen). RNA samples were treated with DNase I to remove potential contaminating genomic DNA, followed by extraction with phenol:chloroform. First-strand cDNA was prepared using oligo (dT) primer. Quantitative PCR was performed on a CFX96 real-time PCR system (BioRad), using iTaq Universal SYBR Green supermix (BioRad). The following cycling conditions were used: 95°C for 30 s, 95°C for 5 s, 60°C for 15 s, and 72°C for 12 s. The reaction was performed for 40 cycles, followed by a step at 72°C for 5 s. Each amplification used 3 technical replicates the results of which were averaged to give the value for a single biological replicate. The primer sequences are provided in Table S1. We tried using the two Arabiodopsis genes (actin and ubiquitin) for qRTPCR, and the results were similar. The data shown in this paper selected Arabidopsis ubiquitin10 (AT4G05320) served as an internal control for normalization.

### Microscopic analysis

Fluorescent and transmission electron microscopy (TEM) were performed using the following protocol. For fluorescent microscopy, the healthy roots or galled roots were treated with Nile red solution (10 μg/ml Nile red in acetone) for 3–5 s, and excess dye was removed after brief rinsing in H_2_O. The root samples were subsequently observed using Nikon fluorescence microscopy. Nile red emits fluorescence over a broad range of wavelengths, but observation using a filter set for B excitation (used for FITC, Cy2, Alexa488, or GFP) generates the best images (Suzuki et al., 2013). For electron microscopy analysis, the roots were fixed in 2.5% glutaraldehyde for 4 h, and were subsequently postfixed in 1% osmium tetroxide for 3 h, washed, dehydrated through an ethanol series, and embedded in London resin white. Ultrathin sections were examined through TEM (HITACHI, H-7000).

### Transverse root sectioning

Wild-type and mutant Arabidopsis were infected with *P. brassicae* for 21 days, and uninfected roots were used as the control. The healthy root tissues and galls were carefully washed with tap water and embedded in a frozen embedding medium (Sakura Finetek USA, Inc., Torrance, CA) at −23°C overnight. Transverse root sections (50 μm thick) were sliced using a freezing microtome (Leica CM1950, Germany) and attached to the slides. A drop of water was added, and the specimens were covered with cover glass. The plant cells and spores were observed using a Nikon light microscope.

### Statistical analysis

Statistical analysis was performed using SPSS (13.0) and a statistics package (Microsoft Excel 2010). In all experiments, One-way ANOVA, specifically Tukey's test with a *P* = 0.05, was used to analyze significant differences between treatment groups.

## Results

### *bik1* exhibited strong resistance to *P. brassicae*

**Figure 1:**
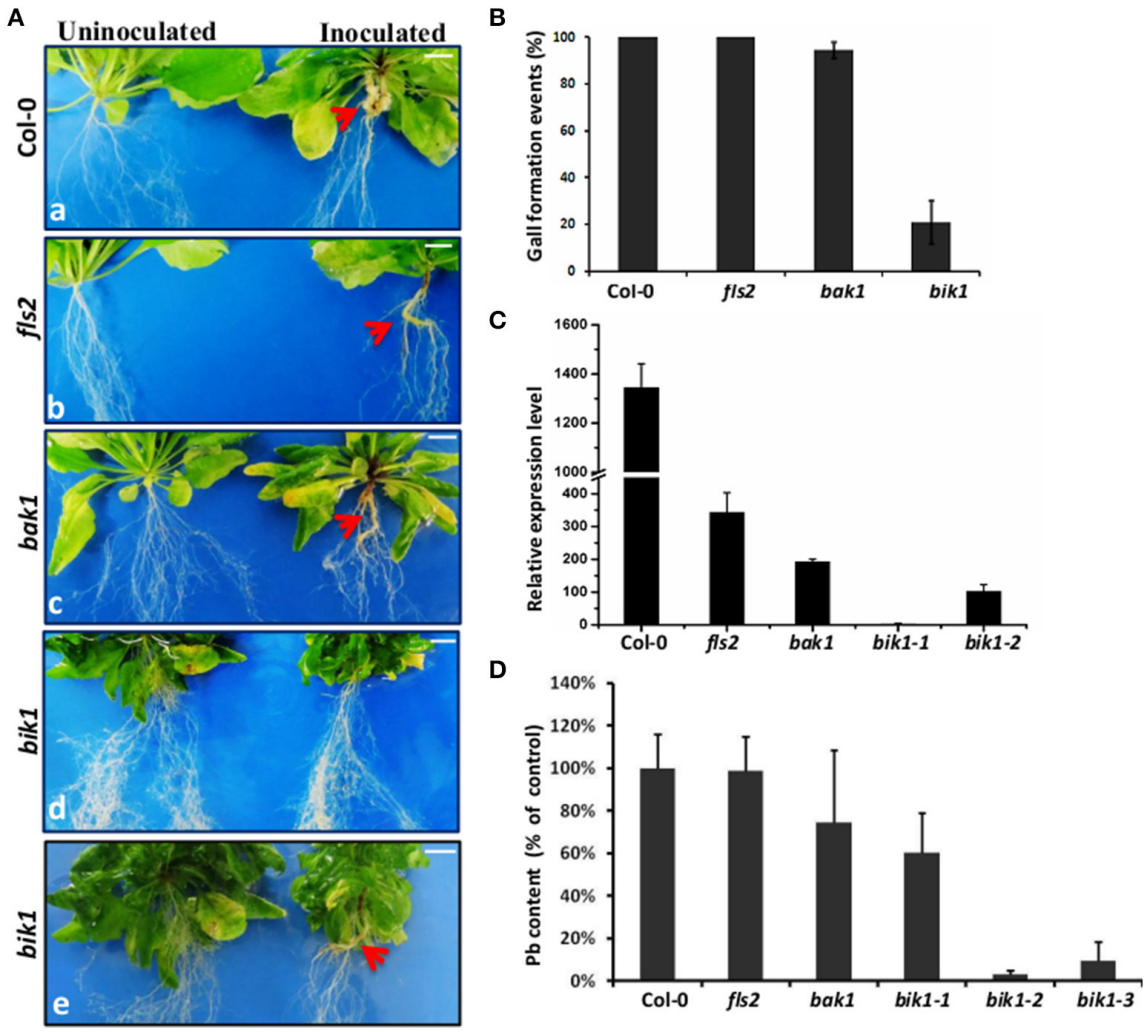
**Phenotypes and ***P. brassicae*** DNA contents in Arabidopsis control and several loss-of-function mutant roots. (A)** Arabidopsis Col-0 roots **(a)** and several loss-of-function mutant RLKs, including FLS2 **(b)**, BAK1 **(c)**, and BIK1 **(d,e)** infected with *P. brassicae*; the root images were captured at 21 days after *P. brassicae* infection. The red arrows show the galls, and *bik1* showed two phenotypes: formation of small galls and no gall formation on the roots. Bar = 0.5 cm. **(B)** Disease symptoms of Arabidopsis control roots and several loss-of-function mutants for gall comparison. Clubroot symptoms were quantified for three biological replicates, each containing 30 plants per genotype. However, for some mutants especially the bik1 mutants several plants were excluded before statistics due to a poor growth (with very small shoots and roots). The error bars represent the SD of the experimental values obtained from three biological replicates. **(C)** Real-time PCR analysis was performed to assess the expression levels of *P. brassicae* at 21 days after inoculation. *bik1-1* showed no root gall formation, and *bik1-2* showed root gall formation. Root sample is a mixture of roots from different plants of the same mutant (over 3 plants). The error bars represent the SD of the experimental values obtained from three technical replicates. **(D)** Pathogen DNA quantification (Pb) by quantitative PCR, expressed the percentage of the mean Pb content in inoculated roots of Col-0, *fls2, bak1*, and *bik1* at 21 days after inoculation with *P. brassicae*. At least 3 plant roots were taken as mixed sample. *bik1-1* showed the formation of small galls on the roots, and *bik1-2* and *bik1-3* showed no formation of galls on the roots. Root sample is a mixture of roots from different plants of the same mutant (over 3 plants). The error bars represent the SD of the experimental values obtained from three technical replicates.

The symptoms of *P. brassicae*-infected plants included the formation of club-shaped galls on the roots, and the wilting, yellowing and premature senescence of the shoots. The plant defense response upon *P. brassicae* infection is often reflected by reduced gall size, root condition, and reduced resting spore production. To determine whether several known receptor-like kinases have function in clubroot-associated defense responses, we observed the infection and colonization of *P. brassicae* on the roots of the loss-of-function mutants (Figure 1). The roots of wild-type Arabidopsis infected by *P. brassicae* were formed of a typical galled, resulting in few rootlets; besides, the infected plants were yellowing (Figure 1A and Figure S3), and the ratio of gall formation was 100% (Figure 1B). The symptoms of *fls2* and *bak1* infected by clubroot-pathogen were similar to those of the wild type (Figure 1A and Figure S3), with a gall formation ratio of nearly 100% (Figure 1B). Interestingly, in *bik1*, the gall formation was inhibited as 79% of the plants that did not form a gall, and the root systems of *P. brassicae*-inoculated *bik1* plants still developed with plentiful lateral roots (Figures 1A,B). The *bik1* mutant line was identified as a homozygous mutant, and the RT-PCR results showed that the *bik1* mRNA could not be detected in homozygous mutant plants (Figure S1), suggesting that these plants were the knockout mutants at the BIK1 locus. To evaluate *P. brassicae* production in the galls, the actin gene expression levels of *P. brassicae* were measured using quantitative real-time PCR. For the phenotype of no gall formation on *bik1*-infected roots due to the largely reduced *P. brassicae* production (by 99.78%), and for the phenotype of mid gall formation, root spore production was reduced by approximately 92% (Figure 1C). The relative amount of *P. brassicae* DNA in total root-extracted DNA was evaluated through quantitative PCR (Figure 1D). The results indicated that root pathogen density was not reduced within the infected roots of *fls2* and *bak1* mutants compared with Col-0, suggesting that *fls2* and *bak1* mutants had no significant change of pathogen density within the root samples. However, the density of *P. brassicae* in the infected roots with and without the formation of small galls in *bik1* mutant was approximately 40 and 90% less than that in Col-0, respectively (Figure 1D). Taken together, the results showed that *bik1* plants exhibited strong resistance to *P. brassicae*.

**Figure 2:**
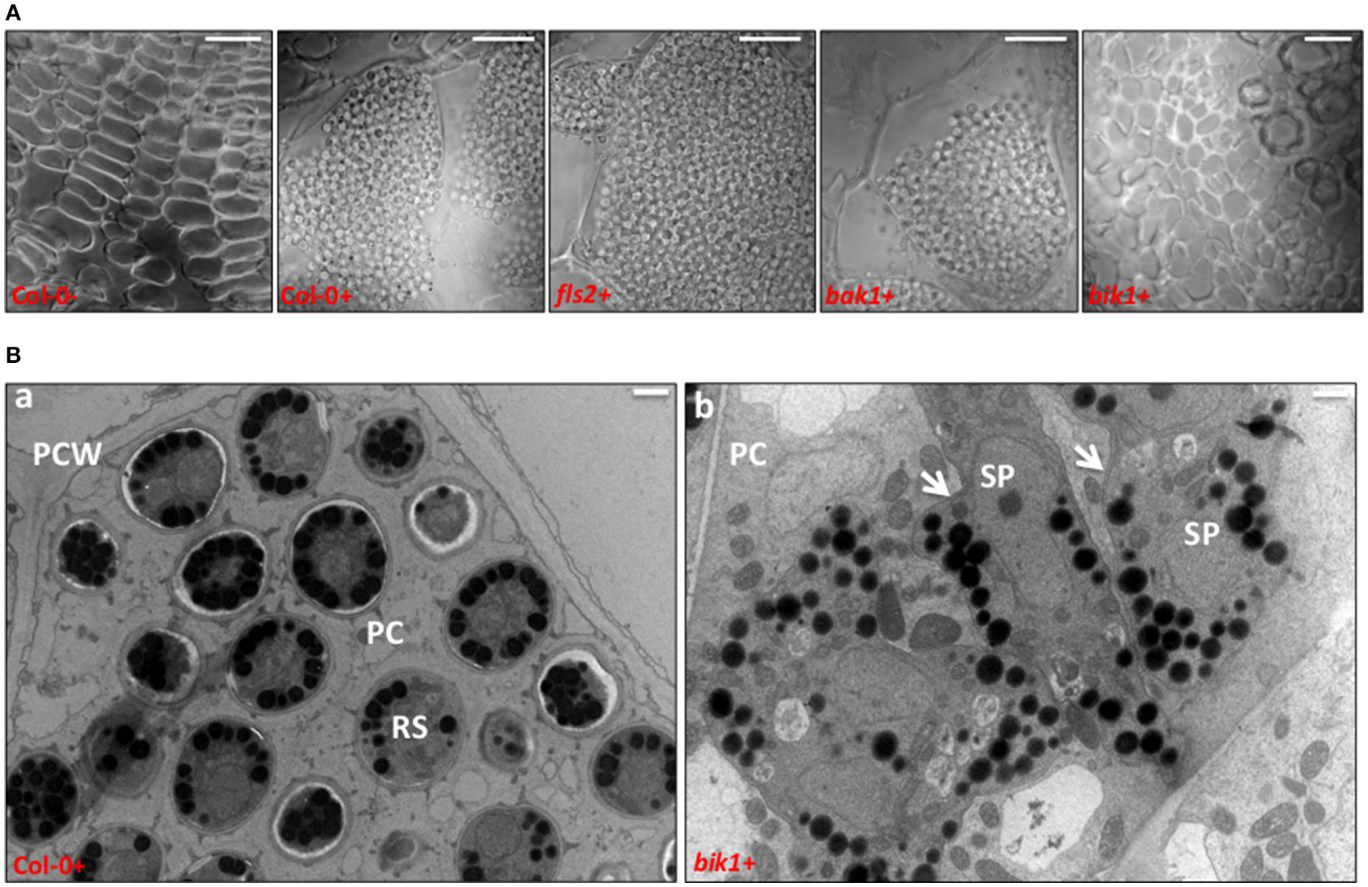
**Longitudinal sections and TEM of Arabidopsis control and loss-of-function mutant roots infected with ***P. brassicae***. (A)** Longitudinal sections of Arabidopsis control and loss-of-function mutant roots infected with *P. brassicae* after 21 days. Bar = 20 μm. **(B)** Transmission electron microscopy of Col-0 **(a)** and *bik1* **(b)** infected *with P. brassicae* for 21 days. Selected clearly visible clubs to section (− = uninoculated roots, + = inoculated roots). RS = resting spores; PC = plant cell; PCW = plant cell wall; SP = secondary plasmodium, white arrow. Bar = 1 μm.

To observe the *P. brassicae* development state in the galls of the infected roots, longitudinal sections of Arabidopsis control and loss-of-function mutant roots were observed. There was no zoospores in the negative control (uninfected wild type Arabidopsis root cells), and many resting spores were found in infected wild type, *fls2* and *bak1* root cells. No obvious zoospores were observed on the infected roots of *bik1*, which showed no gall formation phenotype (Figure 2A). TEM was used to clearly demonstrate the zoospore developmental stages in the very small galls on *bik1* root. The development of *P. brassicae* was in the state of multinucleate secondary plasmodium during cell division on *bik1* root (Figure 2Bb), while the wild-type root cells were filled with resting spores and the cell division was completed (Figure 2Ba). These observations suggested that *bik1* had inhibited development of *P. brassicae*, which explains the results that the wild-type roots showed severe galls with a high production of resting spores, while the *bik1* roots had fewer and smaller galls with less *P. brassicae*.

### Suppression of root hair and cortical infections by the loss of BIK1 function

**Figure 3:**
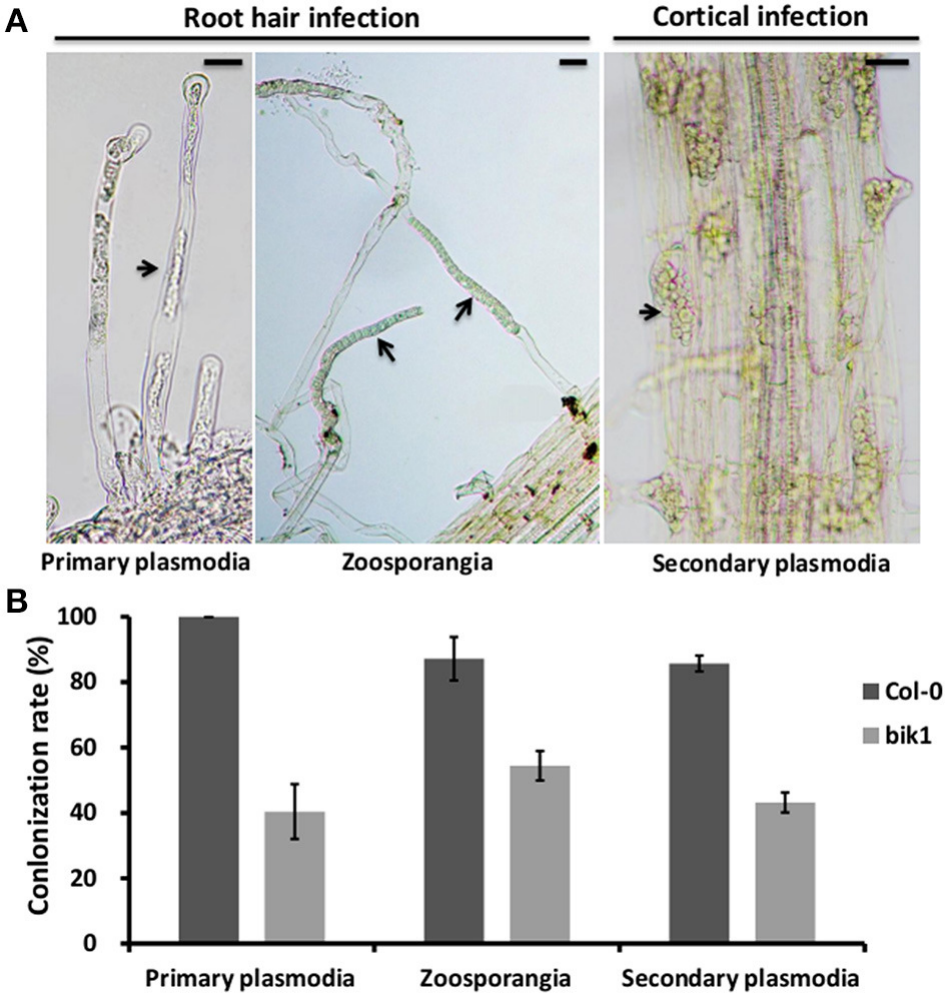
**Root hair and cortical infection stages in the roots of Arabidopsis control and ***bik1*** mutant plants. (A)** The images show the key steps of *P. brassicae* infection in Arabidopsis control and *bik1* mutant plants, including root hair and cortical infections stages. Primary plasmodia of root hair infection were observed in the roots stained with Nile red for 3 days post inoculation. Zoosporangia of root hair infection and secondary plasmodia of cortical infection were observed in the roots stained with Nile red for 12 days post inoculation. The pictures were under light microscopy. Arrows indicate primary plasmodia, zoosporangia or secondary plasmodia. **(B)** Mean rate (100%) of primary plasmodia, zoosporangia and secondary plasmodia per root of the control and *bik1* mutant. The roots of more than 15 plants were selected for the control and *bik1* mutants and sliced into 1–2 cm segments. A total of approximately 100 root segments per sample were observed and counted to determine the presence of infection. The error bars represent the SD of the experimental values obtained from three biological replicates. Bar = 10 μm.

The life cycle of *P. brassicae* consists of three stages: survival in soil, root hair infection, and cortical infection (Schwelm et al., 2015). To investigate the potential involvement of BIK1 in the infection process, we compared the root hair and cortical infections between the wild-type and *bik1 mutant* plants, including the colonization rate of primary plasmodia, zoosporangia, and secondary plasmodia (Figures 3A,B). Nile red was used to label *P. brassicae* for convenient and rapid detection of the stages of infection. The roots of more than 15 plants were selected for the control and *bik1* mutants and sliced into 1–2 cm segments. A total of approximately 100 root segments per sample were observed and counted to determine the presence of infection. Compared with root hair infection of the control (100% primary plasmodia and 87.17% zoosporangia, respectively), the root hair infection ratio of *bik1* mutant roots was reduced, showing 40.40% primary plasmodia and 54.47% zoosporangia, respectively (Figure 3B). Compared with the control roots (85.70% secondary plasmodia), the *bik1* mutant roots showed a reduced cortical infections ratio (43.17% secondary plasmodia) (Figure 3B). Thus, root hair and cortical infections appeared to be impaired by the knockout of BIK1 expression in the mutant.

### High level of ROS accumulated in the infected roots of arabidopsis

**Figure 4:**
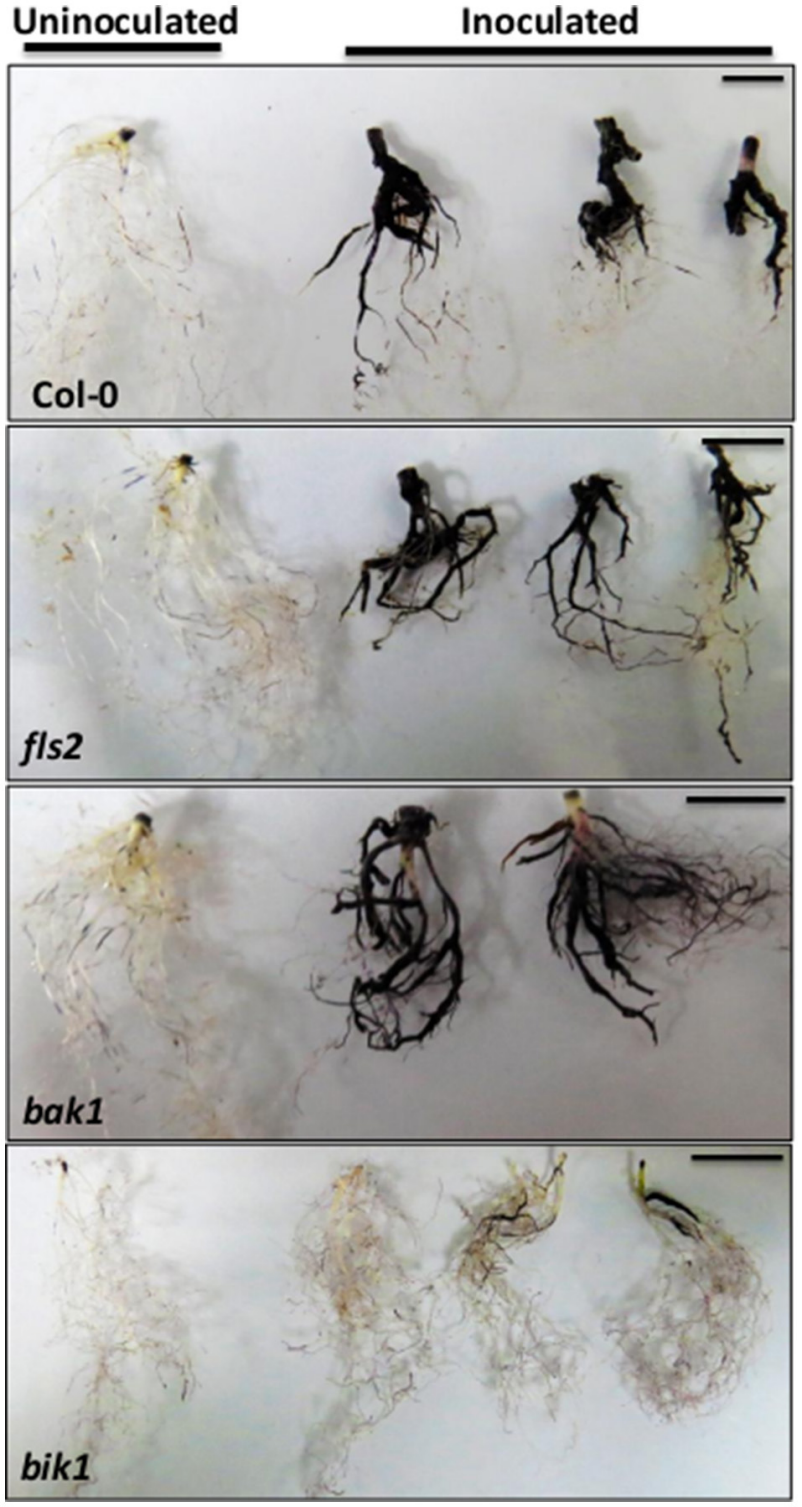
**Analysis of ROS production**. Visualization of ${\text{O}}_{2}^{-}$ production in representative NBT-stained control, and *fls2, bak1* and *bik1* mutant roots at 21 days post inoculation with *P. brassicae*. Dense blue forazan precipitates were observed at the site of H_2_O_2_ production. Bar = 1 cm.

ROS plays dual roles in plants, both as toxic compounds and as the key regulators of many biological processes, such as growth, cell cycle, programmed cell death, hormone signaling, development, stress, and defense responses (Mittler et al., 2004). Pathogen infection may elicit ROS production. We used nitroblue tetrazolium (NBT), a reagent that allows the visualization of superoxide accumulation, to evaluate the ROS production in *P. brassicae*-infected roots of wild type Arabidopsis, *fls2, bak1*, and *bik1* mutants. NBT-formazan precipitates within the roots of all the mutants and control wild type were localized to the places of gall formation (Figure 4), and the NBT-formazan precipitates were much less abundant in the infected roots of *bik1* than in the roots of wild type or *fls2* and *bak1* mutant plants (Figure 4). We further observed the galls of *P. brassicae*-infected *Brassica rapa* through 3-3′-diaminobenzidine (DAB) staining. The results showed that the galled roots had much higher H_2_O_2_ accumulation than healthy roots in the cortex, which was filled with millions of *P. brassicae* resting spores (Figure S4). These results indicated that ROS might be an indicator of defense responses.

### Treatment of roots with SA alleviated the symptoms of clubroot

**Figure 5:**
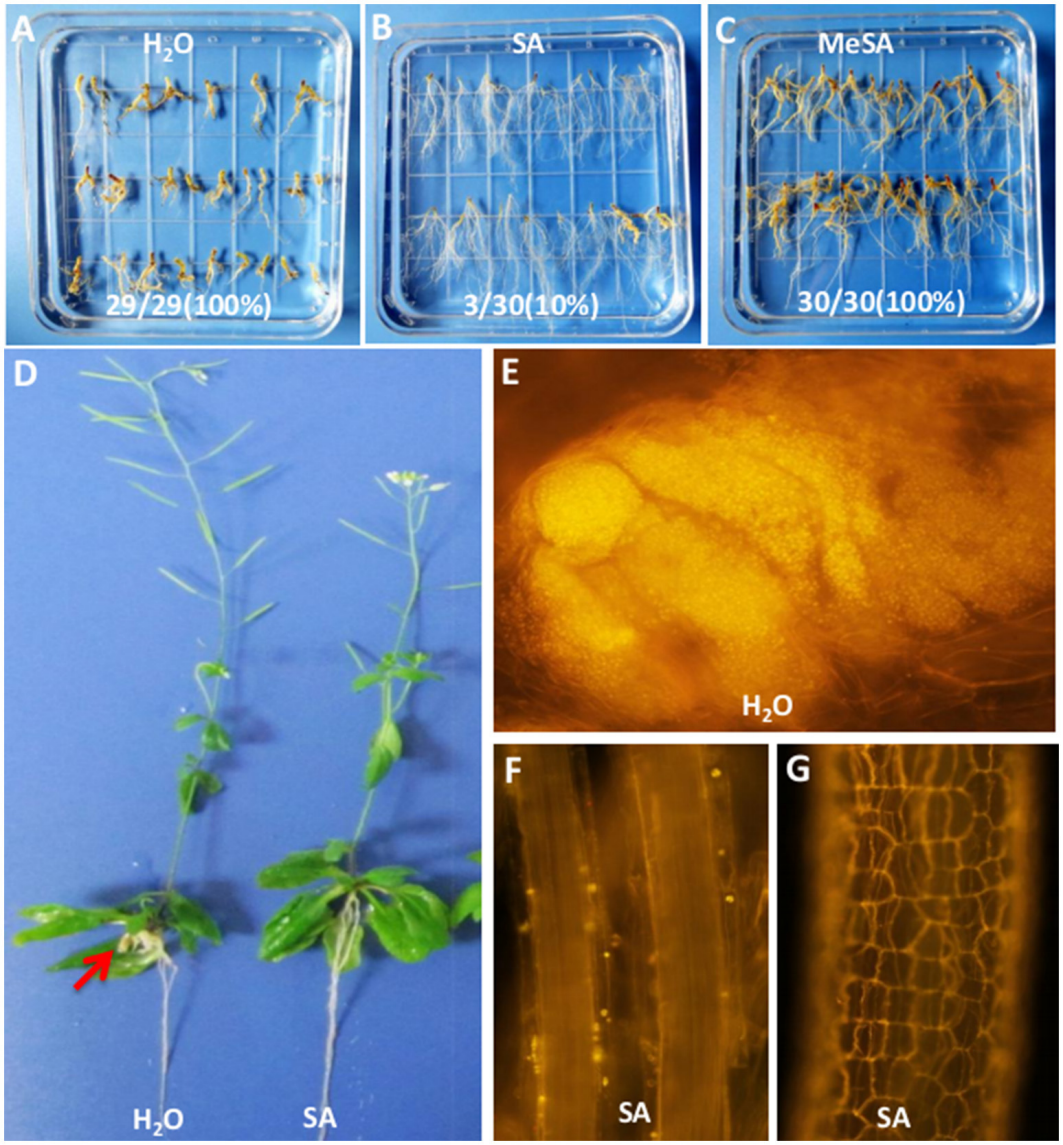
**Effect of SA treatment in the pot on gall reduction in 3-week-old infected plants. (A–C)** Gall phenotype of Arabidopsis Col-0 infected with *P. brassicae* for 21 days in 3 different treatments, including H_2_O (left), SA 2.5 × 10^−5^mol/L for 3 times (middle), and 2.5 × 10^−5^mol/L MeSA for 3 times (right). The number on the picture showed the gall formation events. **(D)** Images of whole plants of Arabidopsis Col-0 infected with *P. brassicae* for 21 days and treated with SA 2.5 × 10^−5^mol/L for 3 times (right) or not (left). **(E)** Nile red staining for infected Col-0 clubroots. **(F,G)** Nile red staining for infected Col-0 clubroots treated with SA.

SA is a central component of defense in plants against a number of biotrophic pathogens (Catinot et al., 2008; Vlot et al., 2009). Thus, we investigated the potential of SA to stimulate defense in Arabidopsis against *P. brassicae*, and methyl salicylate (MeSA) was used as the control. The formation of root galls was assessed at 3 weeks after three repeated treatments with SA or MeSA. SA treatment significantly reduced gall formation, and the proportion of galling was decreased by 90% (from 100 to 10%). MeSA treatment resulted in no reduction of gall formation (from 100 to 100%) (Figures 5A–C). The disease symptoms associated with *P. brassicae* were observed to be decreased in the roots treated with SA, but no observable change was found in shoot morphology (Figure 5D). Nile red staining clearly showed that a very small amount of spores were observed in SA-treated roots (Figures 5E–G). These results indicate that SA enhances the resistance against *P. brassicae*.

### *P. brassicae* altered the expression of defense genes in *bik1*, particularly *PR1*

**Figure 6:**
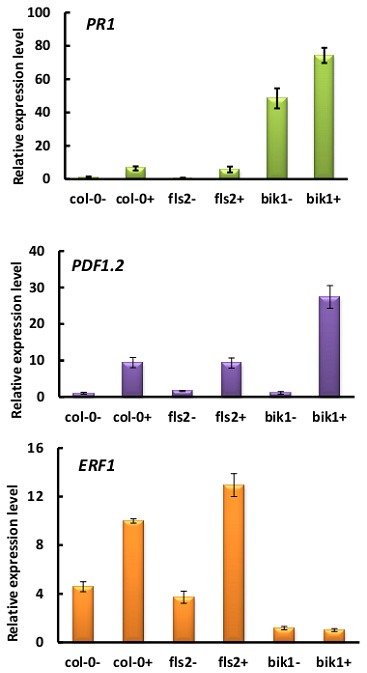
**Relative expression of SA, JA, and ET marker genes ***PR1***, ***ERF1***, and ***PDF1.2*** in response to infection with ***P. brassicae*** for 21 days (− = uninfection, + = infection)**. *P. brassicae* infection up-regulated the expression of these genes in both wild-type and mutant plants.

*P. brassicae*-induced plant defense pathways are often regulated through certain plant hormones, JA pathway, and SA pathway (Lemarié et al., 2015b). The loss of BIK1 function resulted in higher basal SA levels and *PR1* gene expression than wild-type Col-0 Arabidopsis (Veronese et al., 2006; Lei et al., 2014). To determine whether the resistance to *P. brassicae* was conferred through the loss of BIK1 function involving defense-related plant hormones, we detected the expression levels of the SA-signaling marker gene *PR1* and the ET/JA marker genes *ERF1* and *PDF1.2* at 21 days after infection of *P. brassicae* (Figure 6). In wild-type roots, the expression levels of *PR1, PDF1.2*, and *ERF1* were 6.5, 9.4, and 2.2-folds up-regulated after infection, respectively. Similar results were obtained for *fls2* mutant plants. However, the transcript level of the SA-responsive gene *PR1* in *bik1* mutant roots was 48.4-folds higher before *P. brassicae* infection and 11.5-folds higher after the infection compared with that in the wild type roots. These data implied that BIK1 might function as a negative regulator of SA accumulation both in the presence and absence of *P. brassicae* infection.

### *bik1 sid2* double mutant failed to restore the susceptibility to *P. brassicae*

**Figure 7:**
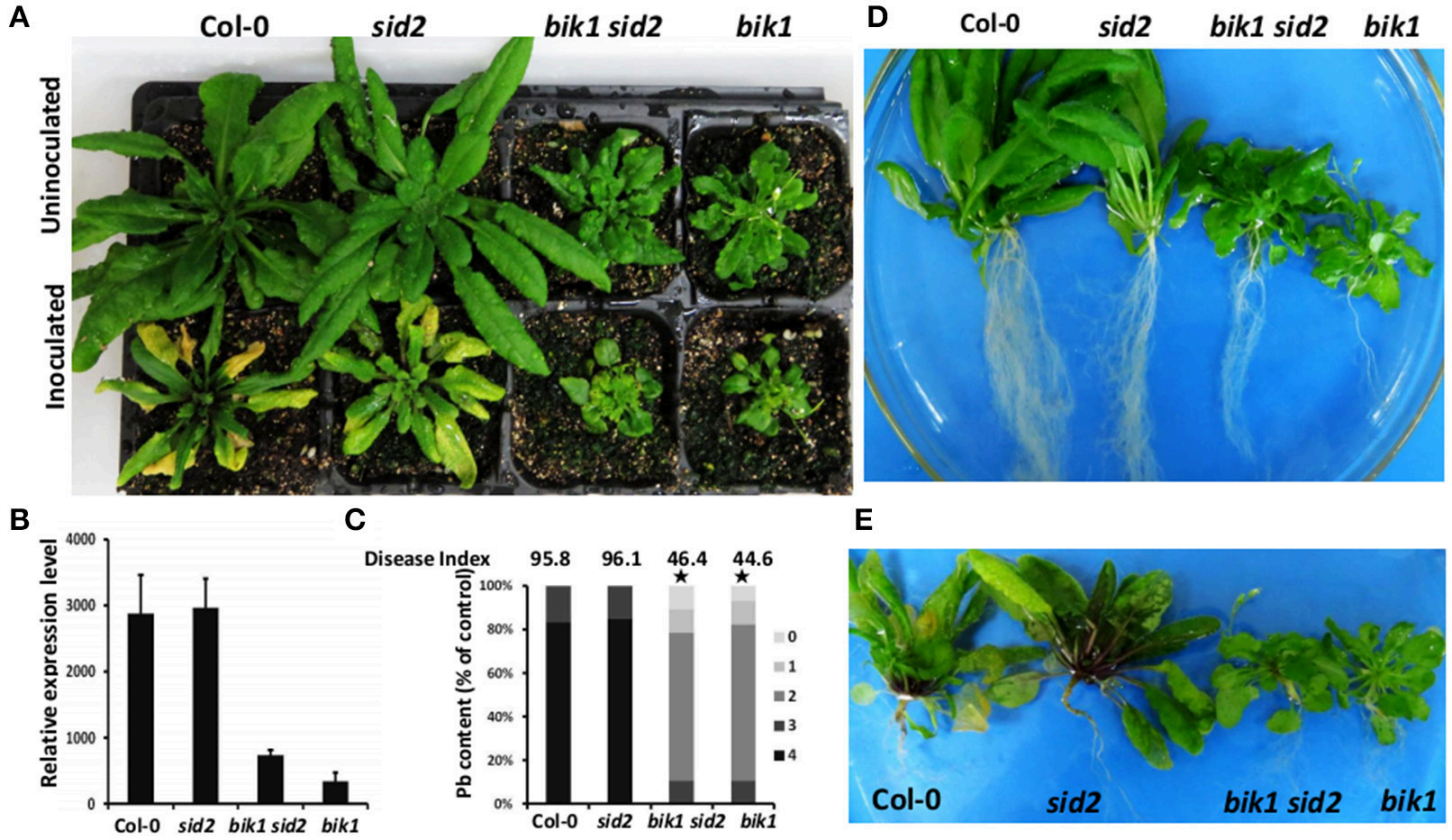
**Effect of ***sid2*** mutation on ***bik1***-mediated resistance against ***P. brassicae***. (A)** Mature shoot phenotypes of wild type and mutant plants. The top of the picture shows uninoculated plants, while the bottom shows plants inoculated with *P. brassicae*. **(B)** Real-time PCR analysis was performed to assess the expression levels of *P. brassicae* at 28 days after inoculation with *P. brassicae*. **(C)** Phytopathological analysis of wild type and mutant plants. The percentages of plants in the individual disease classes are shown. 0, no symptoms; 1, very small galls mainly on lateral roots and that do not impair the main root; 2, small galls covering the main root and few lateral roots; 3, medium to large galls, also including the main root; and 4, severe galls on lateral root, main root or rosette; fine roots completely destroyed. For each treatment, 26–30 Arabidopsis plants were analyzed. The qualitative disease assessment data were initially analyzed using *spss* and subsequently further analyzed after comparing the mean rank differences. The asterisk indicates a significant difference at *P* < 0.01. The disease index for each sample is shown as a number above the respective histograms. **(D)** Images of the shoots without inoculation are shown in **(A)**. **(E)** Clubroot symptoms in plants. Images of the shoots with inoculation are shown in **(A)**.

The loss of SID2 function blocks SA biosynthesis (Wildermuth et al., 2001). To assess the role of SA in *bik1* resistance to *P. brassicae, bik1, sid2*, and *bik1 sid2* double mutant plants were used for infection (Figure 7). The non-inoculated *sid2* mutant did not show any observable change in morphology compared with the wild type (Figures 7A,D, Figure S5). Besides, the severity of clubroot symptoms induced by *P. brassicae* was similar to that of wild-type plants: both of them showed yellowing leaves, severely galled and rotten taproots, and similar density of *P. brassicae* were detected (Figures 7A–C,E). These results are consistent with those reported previously (Lovelock et al., 2016). Apparently, *bik1* reduced the clubroot symptom and density of *P. brassicae* (Figures 7A–E). These results indicated that the elevation of SA accumulation was required for *bik1* resistance. Surprisingly, in the *bik1 sid2* double mutant with reduced SA accumulation (Laluk et al., 2011; Lei et al., 2014), the phenotypes were similar to those of *bik1* plants. These plants showed leaves with serrated margins and wrinkled surfaces, occasional curling, partial recovery to wild type, and strong resistance to *P. brassicae* similarly to *bik1* (Figure 7 and Figure S5). The *bik1 sid2* double mutant line was identified as a homozygous mutant (Figure S2), which showed partial resistance to clubroot possibly due to an epistatic effect or some other unknown signal pathways that contribute to the resistance of *bik1* against *P. brassicae*.

### SAR-deficient mutant *npr1-1* was more susceptible to *P. brassicae* than Col-0

**Figure 8:**
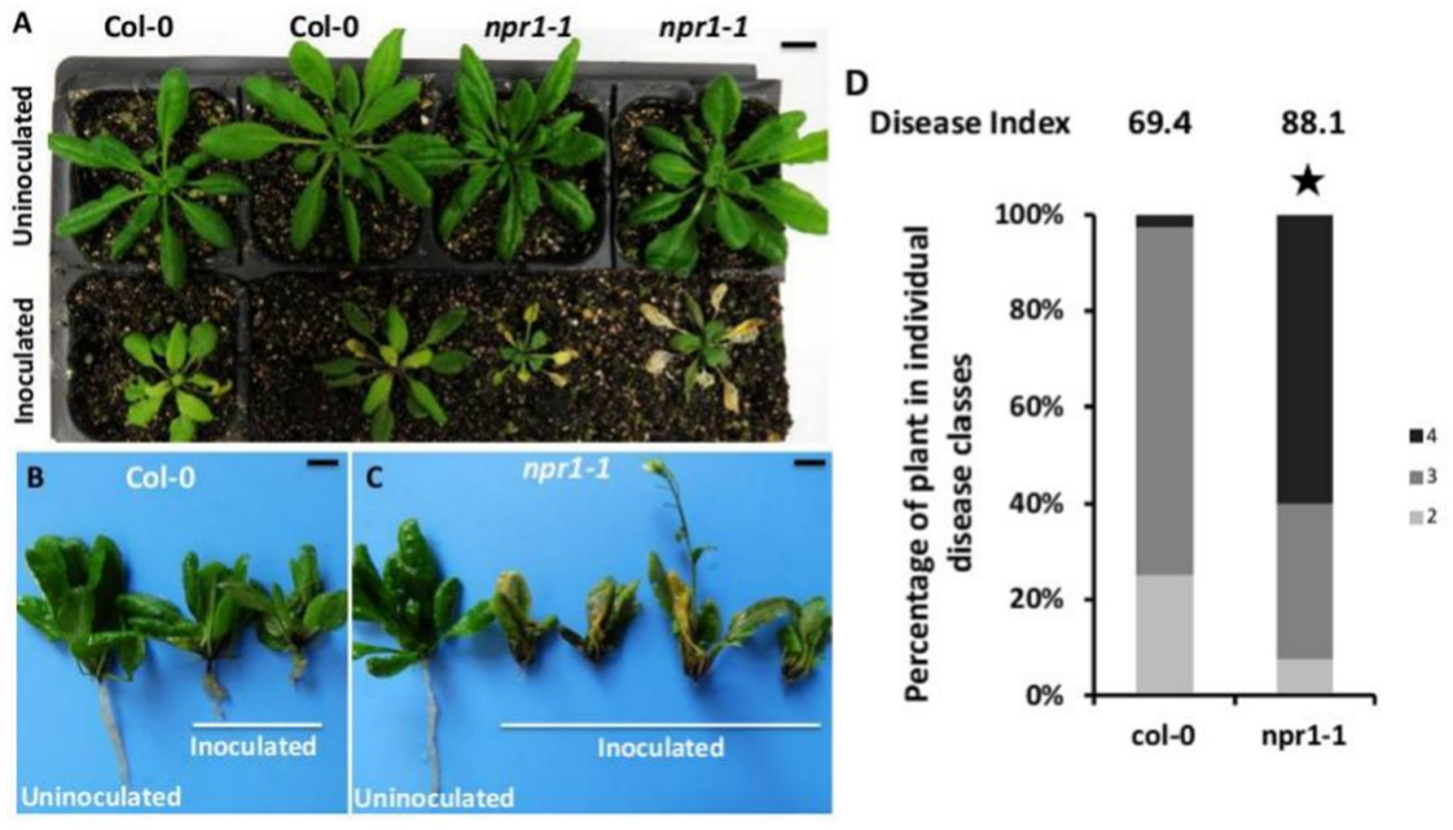
**Gall phenotype and phytopathological analysis of the wild type compared with ***npr1-1*** mutant plants at 21 days post inoculation with ***P. brassicae*****. **(A)** Mature shoot phenotypes of wild-type and mutant plants. The top of the picture shows uninoculated plants, and the bottom of the picture shows plants inoculated with *P. brassicae*. The left two panels show Arabidopsis Col-0 plants, while the right two panels show *npr1-1* mutant plants. **(B)** Gall phenotype of Arabidopsis Col-0. **(C)** Gall phenotype of *npr1-1* mutants. **(D)** Phytopathological analysis of wild type and mutant plants. The percentages of plants in the individual disease classes are shown. For each treatment, 40 Arabidopsis plants were analyzed. The qualitative disease assessment data were initially analyzed using *spss* and subsequently further analyzed after comparing the mean rank differences. The asterisk indicates a significant difference at *P* < 0.01. The disease index for each sample is shown as a number above the respective histogram.

SA and the SA-dependent signaling pathways play a major role in modulating SAR. In Arabidopsis plants defective in SA accumulation, SAR is significantly impaired, and in plants where SA is either over-expressed or externally supplied, enhanced SAR is typically observed (Fu and Dong, 2013). The *bik1* mutant plants showed higher SA level and up-regulated *PR1* gene expression compared with Col-0, indicating that the *bik1* mutant plants had enhanced SAR. To examine whether SAR is important for Arabidopsis to resist *P. brassicae*, the *npr1-1* mutant was inoculated with *P. brassicae*. The results showed that at 21 days post inoculation, the symptoms of *npr1-1* were more severe than those of Col-0 (Figure 8). The infected *npr1-1* leaves turned yellow and showed root rot, while the control plants showed galls on the roots (Figures 8A–C). We then evaluated the disease index in infected Col-0 and *npr1-1* plants (Figure 8D), and the index for Col-0 was 69.4, while that for *npr1-1* was 88.1. These results indicated that *npr1-1* was more susceptible to *P. brassicae* than Col-0. Thus, the SAR of Arabidopsis also contributed to the resistance against *P. brassicae*.

## Discussion

In the present study, we investigated the role of PAMP/MAMP signal receptors in the resistance of Arabidopsis against the infection of clubroot pathogen, and we found that Arabidposis mutant *fls2* and *bak1* were as susceptible to *P. brassicae* as wild-type plants; however, mutant *bik1* displayed strong resistance to *P. brassicae*, and exhibited suppressed root hair and cortical infections. The *bik1* mutant plants were reported to have a higher level of SA than wild-type plants (Veronese et al., 2006; Lei et al., 2014), and real-time PCR proved that the infection of *P. brassicae* induced *PR1* gene expression. *bik1* plants showed higher *PR1* gene expression than Col-0 both in the presence and absence of *P. brassicae* infection. Unsurprisingly, exogenous treatment with SA could enhance the resistance of wild type plants since similar findings were reported by other groups recently (Lovelock et al., 2013, 2016; Lemarié et al., 2015b). However, we found that *bik1 sid2* double mutant with reduced SA accumulation also had strong resistance to *P. brassica*. If it was not due to epistatic effect, the resistance of *bik1* could be attributed to both SA-mediated signal pathway and other unknown factors.

BIK1 is an immediate convergent substrate of several different pattern recognition receptors, including FLS2 (which binds bacterial flagellin), EFR (which binds bacterial elongation factor Tu), PEPR1 (which binds endogenous AtPep peptides), CERK1 (which binds fungal chitin), and BAK1 (which phosphorylates several immune receptors), and is a key component of the plant immune system (Monaghan et al., 2015). Plants lacking functional BIK1 and related proteins such as PBL1 (*bik1 pbl1* mutants) are strongly impaired in PTI signaling and are more susceptible to bacterial and fungal pathogens (Veronese et al., 2006; Lu et al., 2010; Feng et al., 2012). Inactivation of *BIK1* causes severe susceptibility to necrotrophic fungal pathogens *B. cinerea* and *Alternaria brassicicola*, but enhances resistance to biotrophic bacterial pathogen *P. syringae* pv *tomato*. BIK1 acts as a negative regulator of basal resistance to virulent bacterial pathogens (Veronese et al., 2006). BIK1 is highly induced by *B. cinerea* and *P. syringae* (Veronese et al., 2006). Here, we did not detect any significant change in BIK1 (Figure S6A). The strong resistance mediated by *bik1* to aphids depends on the suppression of *PAD4* expression (Lei et al., 2014). *PAD4* was induced in response to aphid feeding, but was significantly reduced in response to clubroot infection (Figure S6B), Thus, BIK1 acts as a negative regulator of the defense response against the infection of *P. brassicae*, and the mechanism may be different from that of the resistance of BIK1 against biotrophic bacterial pathogens and phloem sap-feeding aphids. *P. brassicae* infection alters the phytohormone contents in Arabidopsis, and the plant hormone brassinosteroids (BR) plays a role during gall formation; besides, the BR synthesis inhibitor propiconazole and the BR receptor mutant *bri1-6* reduce gall formation (Schuller et al., 2014). However, phenotypic, molecular, and biochemical data suggested that BIK1 negatively regulates BR-mediated responses and signaling via the phosphorylation through BRI1, and the *bik1* mutant possesses enhanced BR signaling (Lin et al., 2013). These results suggest that resistance to *P. brassicae* in *bik1* mutant is unlikely to be caused by the plant defense-associated hormone BR; on the contrary, the mutant *bik1* needs partial compensation for the negative effect caused by the enhanced BR signaling.

SAR is one mechanism of induced defense that confers long-lasting protection against a broad spectrum of microorganisms (Durrant and Dong, 2004). SA and the SA-dependent signaling pathways play a major role in modulating SAR. In Arabidopsis plants defective in SA accumulation, SAR is significantly impaired, and in plants where SA is either over-expressed or externally supplied, enhanced SAR is typically observed (Fu and Dong, 2013). The Arabidopsis NPR1 is a key regulator in the signal transduction pathway that leads to SAR (Kinkema et al., 2000), and *npr1* mutant fails to respond to various SAR-inducing agents (SA, INA, and avirulent pathogens), displaying little expression of *PR* genes (*PR5* was 5-fold lower and *PR1* was 20-fold lower than the wild type), and exhibiting increased susceptibility to bacterial and fungal infections (Cao et al., 1994). We found that *npr1-1* mutant was more susceptible to *P. brassicae* than wild-type plants, suggesting that SAR might be involved in the defense of plants to deal with the infection and colonization of *P. brassicae*. In dicotyledonous plants, SA is necessary and sufficient SA induces SAR (Vernooij et al., 1994). The *bik1* mutant plants showed higher SA levels and up-regulated *PR1* gene expression compared with Col-0, indicating that SAR was activated in *bik1* mutant plants, which is consistent with the results of Veronese et al. (2006). The *bik1* mutation affects the expression of defense-related genes *ERF1* and *PDF1.2* (Figure 6). These results indicate that *bik1* mutants had some changes in certain plant hormones, including SA, JA, and ET, which is consistent with the results of Veronese et al. (2006). Both the JA and the SA pathways contribute to the resistance against the biotrophic clubroot agent *P. brassicae* in Arabidopsis (Lemarié et al., 2015b). Further studies are needed to examine whether elevated JA and ET signaling in *bik1* plays a role in the resistance against *P. brassicae*.

Root hair infection is the first step for the colonization of *P. brassicae* on host roots. When we examined the infection of *P. brassicae* on *bik1* mutant, the root hair infection and cortical infection were reduced by nearly 50% compared with wild-type plants. This result suggests that the resistance of *bik1* mutant also occurred as early as at primary infection stage. The possible explanation is that BIK1 produces some unknown active compounds to inhibit the infection, or the structure of root hairs is likely to be slightly changed compared with that of the wild-type plants. A previous study showed that the root system of *bik1* was different from that of wild-type plants: *bik1* mutant was rich in root hairs, and its root hairs were longer than those of wild-type plants (Veronese et al., 2006). We also found that the developmental process of *P. brassicae* in cortical cells of *bik1* mutant was significantly suppressed. In the galls of wild-type plants, numerous resting spores were observed, while in the infected-roots of *bik1* mutant, only a few resting spores could be observed, and most of them were plasmodia. Furthermore, based on the quantity analysis of the expression of *P. brassicae* actin gene, the density of *P. brassicae* in *bik1* mutant was significantly reduced. Whether it is SA-meditated signal pathway or other factors that are involved in this suppression of primary infection and the development of *P. brassicae* in cortical cells needs further clarification.

ROS is important for counteracting against the invasion of other organisms. ROS is used to trigger cell death to prevent pathogens from establishing parasitic relationships with their hosts; besides, ROS also could kill pathogens directly. As a biotrophic pathogen, *P. brassicae* depends on host living cells for nutrients and proliferation. In this study, we found high level ROS accumulation at the place where *P. brassicae* colonized in the roots of wild-type plants, *fls2* and *bak1*, while relatively low level of ROS accumulation was observed in the roots of *P. brassicae*-inoculated *bik1*. These results suggest that oxidative stress induced by clubroots is an important immune response against invasion of pathogens. However, it is still largely unknown whether these ROS are generated by the host or by *P. brassicae*. NADPH oxidase (respiratory burst oxidase homologs) genes can be found in the P. *brassicae* genome; by analyzing the transcripts within specific life stages, NADPH oxidase genes were found to be expressed at every stage of development. We may use Arabidopsis knockout mutants without ROS production or with ROS overproduction to investigate the role of ROS during *P. brassicae* infection in th future.

## Author contributions

TC and DJ designed research; TC, KB, ZH, ZG, and YZ performed research; TC, YF, JC, JX, and DJ analyzed data; TC and DJ wrote the paper. All authors read and approved the final manuscript.

## Funding

This work was supported by the earmarked fund for China Agriculture Research System (CARS-13), the Fundamental Research Funds for the Central Universities (2013PY115), Natural Science Foundation of Hubei Province of China (2013CFB202) and the Specialized Research Fund for the Doctoral Program of Higher Education of China (20130146120032).

### Conflict of interest statement

The authors declare that the research was conducted in the absence of any commercial or financial relationships that could be construed as a potential conflict of interest.

## Supplementary material

Figure S1 **Phenotype and identification of the homozygous *bik1* mutant. (A)** Phenotype of 5- **(a,b)** and 10-week-old **(c)** Arabidopsis plants and *bik1* mutants. **(B)** Identification of the homozygous *bik1* mutant. **(a)** Diagram showing primer and T-DNA insertion sites. Genomic PCR **(b)** and RT-PCR **(c)** of homozygous *bik1* lines. Ubiquitin PCR product was used as an internal control.

Figure S2 **Identification of the homozygous *bik1 sid2* double mutant**. Genomic PCR **(A)** and RT-PCR **(B)** of homozygous *bik1 sid2* lines. Ubiquitin PCR product was used as an internal control. Primer bik1(3) indicates the 3 primers used once, BIK1-LB, BIK1-LP, and BIK1-RP. Primer sid2 (3) indicates the 3 primers used once, SID2-LB, SID2-LP, and SID2-RP.

Figure S3 **Mature shoot phenotypes of various Arabidopsis genotypes used in the present study**. Shoot images were acquired at 21 days after *P. brassicae* infection. Bar = 2 cm.

Figure S4 **DAB staining for production of H_2_O_2_ in *P. brassicae*-inoculated plants**. Production of H_2_O_2_ in mock-inoculated **(A–C)** or *P. brassicae*-inoculated **(D–E)** plants at 21 days after infection. The roots were stained with DAB as described in the Methods section. **(A,D)** Were root phenotype, bar = 5 mm. **(B,E)** Were the root slices, the red arrows showed the resting spores, bar = 20 μm. **(C,F)** were the roots stained with DAB. The brown precipitate shows DAB polymerization at the site of H_2_O_2_ production, Bar = 20 μm. The experiments were repeated three times with similar results.

Figure S5 **Phenotypes of various Arabidopsis genotype mutants**. Growth morphology of Arabidopsis wild type and mutant plants in the present study without infection with *P. brassicae*.

Figure S6 **Relative expression of *BIK1* (A) and *PAD4* (B) in wild type with or without *P. brassicae* inoculation for 21 days**. (− = uninoculated roots, + = inoculated roots).

Table S1 **Primers used in this study**.
