# Supplementary figures and images for: Arabidopsis Mutant bik1 Exhibits Strong Resistance to Plasmodiophora brassicae
Source: Front Physiol. 2016 Sep 13;7:402. doi: 10.3389/fphys.2016.00402 (PMC5020103; doi:10.3389/fphys.2016.00402)

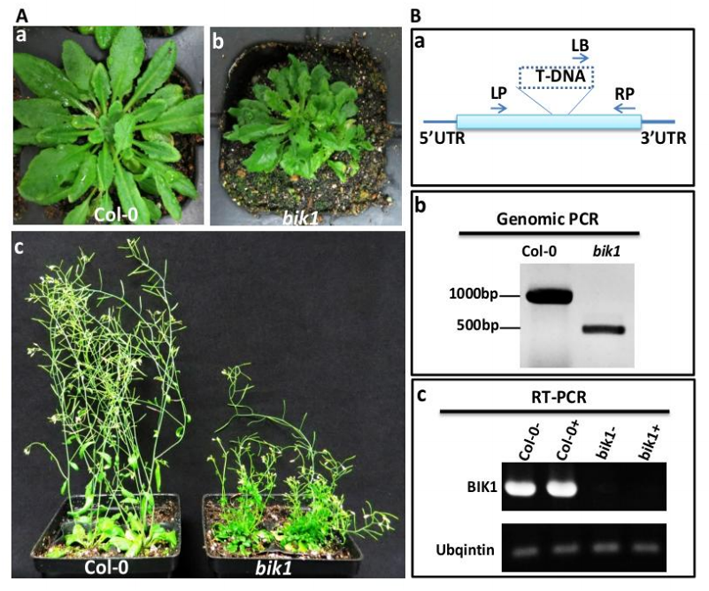

Supplement: Figure S1 — Phenotype and identification of the homozygous bik1 mutant. (A) Phenotype of 5- (a,b) and 10-week-old (c) Arabidopsis plants and bik1 mutants. (B) Identification of the homozygous bik1 mutant. (a) Diagram showing primer and T-DNA insertion sites. Genomic PCR (b) and RT-PCR (c) of homozygous bik1 lines. Ubiquitin PCR product was used as an internal control. [file Image1.TIF]

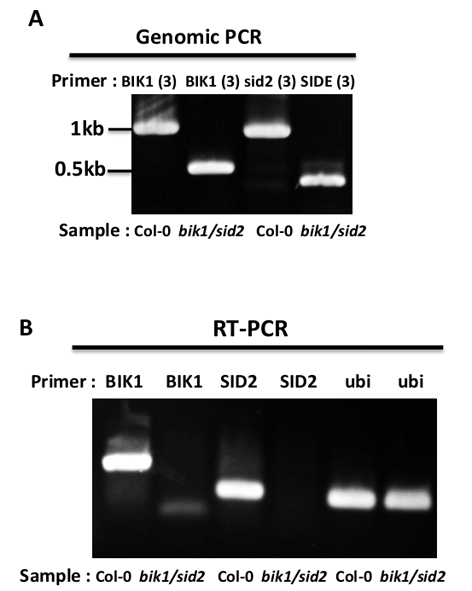

Supplement: Figure S2 — Identification of the homozygous bik1 sid2 double mutant. Genomic PCR (A) and RT-PCR (B) of homozygous bik1 sid2 lines. Ubiquitin PCR product was used as an internal control. Primer bik1(3) indicates the 3 primers used once, BIK1-LB, BIK1-LP, and BIK1-RP. Primer sid2 (3) indicates the 3 primers used once, SID2-LB, SID2-LP, and SID2-RP. [file Image2.TIF]

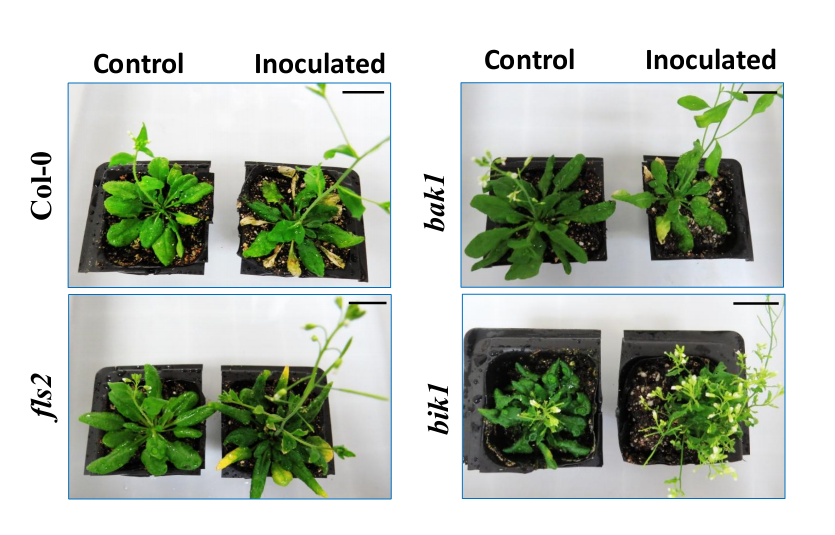

Supplement: Figure S3 — Mature shoot phenotypes of various Arabidopsis genotypes used in the present study. Shoot images were acquired at 21 days after P. brassicae infection. Bar = 2 cm. [file Image3.TIF]

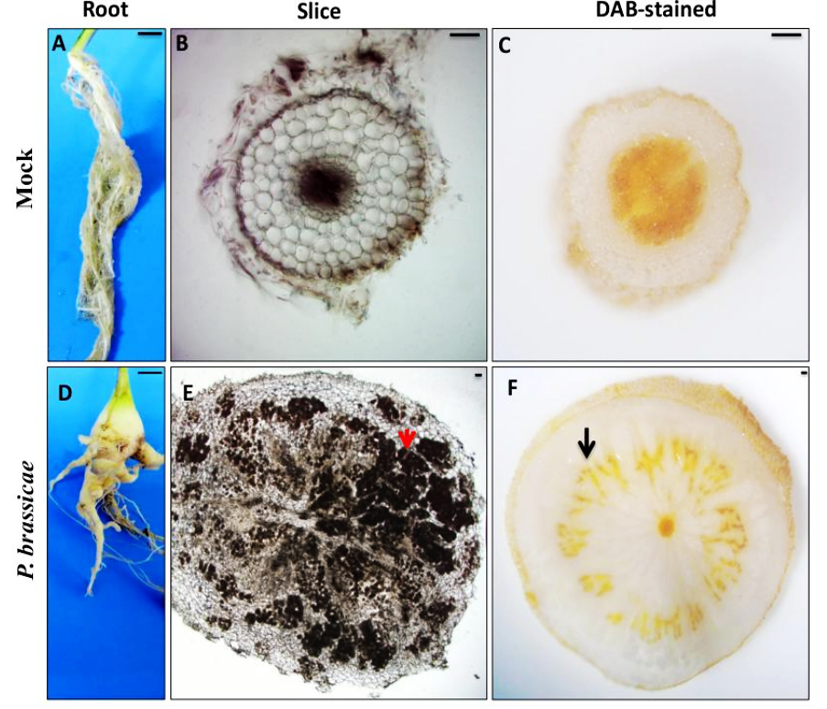

Supplement: Figure S4 — DAB staining for production of H2O2 in P. brassicae-inoculated plants. Production of H2O2 in mock-inoculated (A–C) or P. brassicae-inoculated (D–E) plants at 21 days after infection. The roots were stained with DAB as described in the Methods section. (A,D) Were root phenotype, bar = 5 mm. (B,E) Were the root slices, the red arrows showed the resting spores, bar = 20 μm. (C,F) were the roots stained with DAB. The brown precipitate shows DAB polymerization at the site of H2O2 production, Bar = 20 μm. The experiments were repeated three times with similar results. [file Image4.TIF]

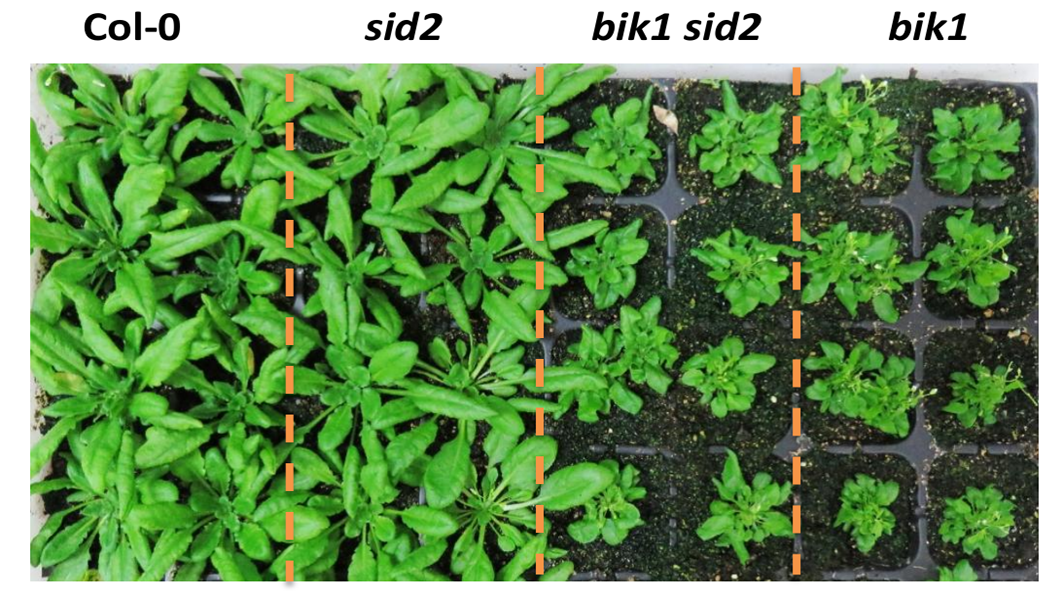

Supplement: Figure S5 — Phenotypes of various Arabidopsis genotype mutants. Growth morphology of Arabidopsis wild type and mutant plants in the present study without infection with P. brassicae. [file Image5.TIF]

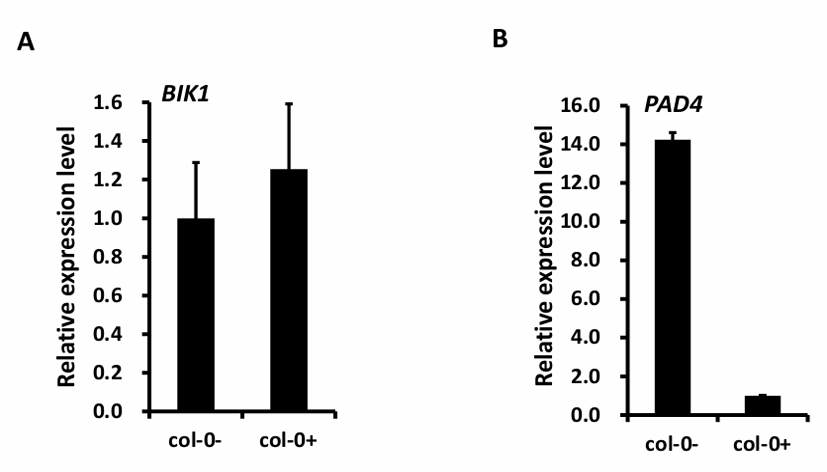

Supplement: Figure S6 — Relative expression of BIK1 (A) and PAD4 (B) in wild type with or without P. brassicae inoculation for 21 days. (− = uninoculated roots, + = inoculated roots). [file Image6.TIF]
